# Radiofrequency Ablation for Post Infarction Ventricular Tachycardia

**Published:** 2004-04-01

**Authors:** David O'Donnell, Voltaire Nadurata

**Affiliations:** Electrophysiology Unit, Austin Hospital, Heidelberg, Melbourne Australia

## Abstract

Radiofrequency ablation has an important role in the management of post infarction ventricular tachycardia. The mapping and ablation of ventricular tachycardia (VT) is complex and technically challenging. In the era of implantable cardioverter defibrillators, the role of radiofrequency ablation is most commonly reserved as an adjunctive treatment for patients with frequent, symptomatic episodes of ventricular tachycardia. In this setting the procedure has a success rate of around 70-80% and a low complication rate. With improved ability to predict recurrent VT and improvements in mapping and ablation techniques and technologies, the role of radiofrequency ablation should expand further.

## Introduction

Radiofrequency ablation (RFA) is the treatment of choice in the management of symptomatic patients with ventricular tachycardia (VT) in the absence of structural heart disease. The role of RFA in-patients with VT in the setting of chronic ischemic heart disease is less well defined. Ventricular tachycardia (VT) is a common complication of ischemic heart disease, with significant associated morbidity and mortality [1,2]. Traditionally, anti-arrhythmic medications have formed the mainstay of treatment, despite the low efficacy, the risk of pro-arrhythmia and long term adverse effects [3]. Anti-arrhythmic surgery is successful in abolishing VT, however, the operative mortality in most series was unacceptably high [4]. Over the past 10 years, implantable cardioverter defibrillators (ICDs) have become the treatment of choice for all but incessant VT. This treatment is based on the evidence of a number of studies which have shown that ICDs reduce overall mortality in certain subgroups of patients with ischemic heart disease with documented VT [53,6], or even in the absence of previously documented VT [7].

The development of anti-tachycardia pacing algorithms has significantly reduced the need for cardioversion [8] and resulted in much improved patient acceptance of such devices. Whilst ICDs are associated with significant mortality benefits in-patients with ischemic heart disease and VT, they do not actually prevent the onset of arrhythmia and remain relatively contraindicated in-patients with frequent arrhythmic episodes. In addition the implantation and function of an ICD can contribute to pathological anxiety and depression in some patients [9]. Furthermore, ICD therapy is relatively expensive, when considered over the expected lifetime of particularly younger patients. In-patients with ischemic heart disease RFA has developed predominantly an adjunctive role in patients with incessant or highly symptomatic, drug refractory VT.

## Defining the Substrate for ventricular tachycardia in ischemic heart disease

VT in the setting of ischemic heart disease is predominantly manifest as monomorphic VT, caused by stable reentrant circuits [10,11]. Whilst polymorphic VT and primary ventricular fibrillation are seen in patients with ischemic heart disease these more often relate to acute biochemical, ischemic or pharmacological insults; whilst ablation has been proposed for these arrhythmias [12], correction of the underlying abnormalities form the first line of treatment.

In simple terms the mechanism of reentry in ischemic heart disease relates to zones of heterogeneous conduction, typically at the edge of myocardial scars. Residual functioning myocytes at the edge of, or interspersed within, myocardial fibrous scar create zones of slow conduction and conduction block [13]. When appropriately located and with appropriate electrophysiological properties, these slow conduction zones can take the form of a discrete electrical channel or isthmus critical to the initiation and continuation of VT.

**Figure 1:**
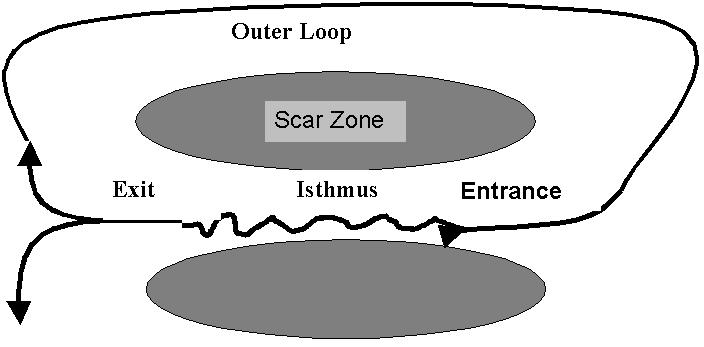
Schematic representation of the re-entrant circuit of ventricular tachycardia. The circuit has a central critical isthmus located in a position intimately related  to the scar border zone. The isthmus can be formed between the scar zone and normal myocardium, within or between two scar zones or between the scar zone and an anatomical boundary such as the mitral valve. The electrical activation travels slowly through the isthmus before breaking out into normal myocardium at the exit zones. The circuit courses a number of possible routes (outer loops, inner loops or both) before returning to the critical isthmus at the entrance zone.

The re-entrant VT circuit utilizes the slow conduction properties of the critical isthmus. (Figure 1) The electrical activity propagates relatively slowly along the isthmus during diastole and forms a silent zone on the surface ECG. When electrical activity reaches the exit zone it rapidly propagates throughout the ventricular myocardium, giving rise to mechanical systole and the QRS complex on the surface ECG. The electrical wavefront courses a loop through the myocardium before returning to the entrance zone and repeating the isthmus passage [14].

The diagrammatic representation of re-entry in Figure 1 is a simplistic description of a process, which can be highly complex. The reentry circuits can be multiple with shared and separate components; the circuits can be located in the endocardium, epicardium, transmuraly or throughout each of these zones. The isthmuses can vary significantly in length and electrophysiological properties. The regions of conduction block can be formed by scar tissue or by anatomical boundaries (typically mitral annulus) and can manifest absolute conduction block or functional conduction block developing only during VT. A comprehensive knowledge of the anatomy and electrophysiology is fundamental to approaching ablation of VT in ischemic heart disease.

## Mapping techniques

Electrophysiological mapping techniques are primarily focused to identify appropriate sites for ablation.  In the mapping of VT in the setting of ischemic heart disease, the fundamental task is to identify and understand the critical VT isthmus. A number of different techniques have been developed to guide mapping.

## Mapping During VT

### Early systolic Activity

**Figure 2:**
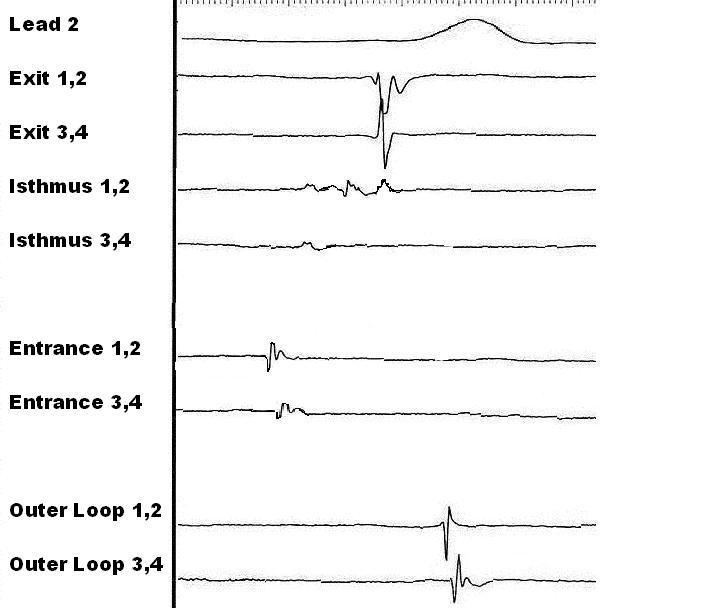
Intra-cardiac electrogram recordings from a single patient with typical re-entrant ventricular tachycardia related to a previous inferior infarction. The recordings were obtained sequentially and superimposed. The exit zone is identified by a discrete electrogram, which occurred 33 msec before the onset of the surface QRS. Pacing at this site in sinus rhythm produced a QRS complex, which closely resembled that of the studied VT. The common isthmus is identified by a slow moving, complex, fractionated signal in late diastole. This position was less than 1 cm from the exit site. Pacing at this site during VT (with high local capture threshold) resulted in concealed entrainment, with a post pacing interval of less than 20 msec different from the VT cycle length. Pacing in sinus rhythm produced a complex similar to the VT being studied and a long stimulus to QRS onset. A complex late potential was seen at this site in sinus rhythm. The entrance zone and outer loop contained discrete local potentials, which were seen early in diastole and mid QRS respectively. Radiofrequency ablation using an irrigated tip catheter applied at the site of the isthmus recording terminated VT. The local lesion was consolidated with a linear ablation encompassing the exit zone and extending to the mitral annulus. Following this lesion this VT morphology was no longer inducible.

Traditional mapping approaches during VT have focused on identification of earliest systolic activation. The principle is that the electrical activation that precedes the QRS complex represents the breakout point of the VT into relatively normal myocardium. Fast moving discrete activity preceding the surface QRS by at least 30 msec is regarded as early activation. As seen in Figure 2, the activity preceding the surface ECG will be recorded at the exit zone. Whilst identification of the exit zones is important, ablation here alone is rarely sufficient to eliminate the re-entry circuit.

### Diastolic potentials

Slow moving activity during diastole can be identified in the critical isthmus during VT [11,15]. The timing of this activity will become progressively earlier as the mapping moves from the exit to the entrance. Whilst this is often a useful finding, alone it is inconsistent; blind loops and far field potentials can give rise to diastolic activity that is unrelated to the critical isthmus. The diastolic potentials which arise in the critical isthmus will remain associated with VT during entrainment mapping (described below), whilst diastolic potentials arising from bystander areas should be dissociated from the VT during entrainment [15]. The morphology of the diastolic activity has also been studied to predict the location of the critical isthmus but has limited additional value.

### Entrainment Mapping

Entrainment refers to a specific response of an arrhythmia to a pacing stimulus at rates greater than the tachycardia cycle length [16]. Whilst no one technique alone can fully identify the re-entrant circuit in is entirety, entrainment mapping is perhaps of most use in localizing the critical VT isthmus [11] [17]. The principles of entrainment utilize the unique electrophysiological properties, which lead to the establishment of a reentrant arrhythmia with an excitable gap. Whilst a complete discussion of entrainment is beyond the scope of this paper, the principles warrant discussion.

Pacing that leads to classical entrainment will result in temporary QRS fusion. If pacing is conducted from within the critical isthmus of the arrhythmia, the resultant QRS morphology should match that of the VT being studied; this is known as entrainment with concealed fusion or concealed entrainment. During concealed entrainment, the duration from the pacing stimulus to the surface QRS onset can localize the pacing site within the isthmus. The shortest S-QRS will be recorded at the exit zone and this will progressively lengthen as the site of pacing moves along the isthmus towards the entrance zone.

When pacing at a site within the re-entry circuit, the post pacing interval, from the last pacing stimuli to the return electrogram seen at the pacing site should be similar to the cycle length of the VT. (Post pacing interval - VT cycle length less than 30 msec.) With the complexity of the reentry circuits, neither concealed entrainment nor the post-pacing interval are completely accurate in identifying the isthmus zone [18]. The utilization of entrainment mapping is further limited by a number of factors including: the dependence upon haemodynamically tolerated rhythms to enable mapping, the ability to capture the local myocardium, which can be difficult in zones of scar with high capture threshold and the often complex and fractionated signals within the scar border zone which complicate interpretation.

## Mapping During Sinus Rhythm

Mapping during VT can be limited by haemodynamic instability, difficulties in inducing VT and uncertainties regarding the clinical significance of induced VTs. Mapping during sinus rhythm is designed to delineate the anatomical and electrophysiological substrate, which predispose to reentry and VT. Mapping in the area of previous infarction can identify these potential areas of abnormal conduction.

Different groups perform substrate mapping using a combination of different techniques. Analysis of electrograms during sinus rhythm can identify regions of fractionated electrograms and late potentials, which are markers of abnormal, slowed conduction. Voltage mapping identifies scar zones and the scar border zones in which most isthmuses are located. Pacing threshold maps add additional information to the voltage map to identify electrically heterogeneous areas. Pacemapping to produce a QRS morphology that is similar to that during VT can roughly localize the site of the reentry circuit exit. The duration from the pacing stimulus to the onset of QRS (S-QRS duration) can further identify abnormal substrate that predisposes to reentry.

None of these above techniques have adequate specificity to be used alone, but in combination can successfully identify regions of abnormal conduction. Substrate mapping does not identify a discrete point for ablation, but rather a zone or region of abnormal myocardium with the potential to facilitate reentry.

## Newer Mapping Tools

The newer mapping tools utilized in electrophysiology all have the potential to facilitate the mapping and ablation of VT. By providing reproducible anatomical information the mapping tools each have the ability to simplify mapping approaches and to guide the delivery of optimal radiofrequency ablation lesions.

CARTO (Biosense-Webster, Diamond Bar, USA) is the most studied mapping tool to aid ablation of ventricular tachycardia. CARTO is most useful in providing accurate substrate maps. The EnSite 3000 multielectrode array (Endocardial Solutions, St Pauls, USA) uses non-contact virtual electrograms which can not only provide myocardial substrate information, but can map ventricular activation with a little as one recorded cardiac cycle.

As technology improves, the mapping systems will become an integral part of the study of ventricular tachycardia. In the foreseeable future, however, the mapping tools will provide guidance and accuracy, but will not replace the need for an operator with a full grasp of electrophysiology and anatomy.

## Ablation Techniques

The ablation of VT in the setting of ischemic heart disease has been performed according to two basic strategies. The first strategy involves induction of ventricular tachycardia, mapping and targeted ablation [19-27]. The second strategy involves substrate mapping in sinus rhythm and linear ablation to eradicate regions of abnormal myocardium with the potential to facilitate reentry.28 It is difficult to comment on the relative success of the different approaches as most centers now use a combination of these two approaches.

Targeted ablation of a single morphology of haemodynamically stable VT can be successful in over 80% of cases [19-27]. Unfortunately, acute procedural success does not always predict long term recurrence of VT. Some studies have reported a recurrence of VT of close to 50% if a single dominant morphology is successfully ablated [25]. As such the trend in ablation of VT has been to target all inducible morphologies of monomorphic sustained VT. When multiple morphologies of VT are targeted the acute success rate diminishes proportional to the number of different morphologies and diminishes further when the VT is hemodynamically unstable [26,29].  However, even in-patients with more than 3 inducible morphologies of VT or with hemodynamically unstable VT procedural success rates are approach 60% [26].

The linear approach pioneered by Marchlinski [28] was acutely successful in eradicating or modifying VT in 7 of 9 patients with a recurrence in less than 20%. Using a combination approach large series report an acute success rate of 60-80%, with a longer-term freedom from VT of 75-95% in those successfully ablated. Recent developments in catheter technology have led to the use of irrigated tip catheters, which enable deeper lesions to be formed. These catheters have been shown to be more successful in the acute termination of VT [30], long term data is not yet available. Alternative approaches via the epicardium [31] and via the coronary arteries [32] have also been used but as yet are not applicable beyond a few expert centers.

## Complications

In the larger reported series, significant complications (cardiac tamponade, TIA, complete heart block) occurred in 5 - 10% of patients and minor complications (access site hematoma, nausea etc) in a further 5 - 10% of patients [19-29].

## Defining success

Previous series have reported the recurrence of VT following a successful procedure to vary from15 - 40% [19-25], depending upon the specific definitions used. The non-inducibility of clinical VT is often regarded as a marker of success following RFA. However, it has been shown that even following successful ablation of all clinical VTs, the presence of inducible non-clinical VT is associated with an increased incidence of recurrent VT [21]. Indeed, following ablation of all morphologies of clinical VT in one series, further morphologies, which had not been documented clinically, were inducible in 83% of patients [26].

Because of the difficulties with recognizing and defining clinical VT, we prefer to define success based on the level of stimulation required to induce VT rather than the particular morphology of VT induced [33]. Prior to ablation, we conduct a stimulation protocol from the right ventricular apex comprising an 8 beat drive followed by up to 5 extrastimuli introduced at decreasing cycle lengths until refractoriness was reached. At the end of the ablation procedure we repeat the stimulation protocol. Procedural success was defined by comparing the results of the two stimulation protocols.

- *Acute success* - No monomorphic VT induced at refractoriness of 5 extra stimuli.
- *Modified Result* - Monomorphic VT only inducible by 2 extra stimuli more aggressive than at baseline.
- *Failed Procedure* - Monomorphic VT inducible at similar levels to baseline.

Using this protocol we were able to accurately predict the patients who will remain free of further VT following radiofrequency ablation. In patients with a minimum follow up of 12 months the rate of recurrent VT with either a complete success or a modified result was 4%, compared with 66% amongst patients with a failed procedure [33].

## Patient selection

At one end of the spectrum, the patient with incessant or intractable ventricular tachycardia, resistant to pharmacotherapy and overdrive pacing and requiring frequent cardioversion is an obvious candidate for ablation of ventricular tachycardia. At the other end of the spectrum there is presently no role for ablation in the sense of primary prevention.

Patients with frequent episodes of VT should be considered for ablation. The threshold for deciding to undergo ablation should consider the local expertise, the frequency of episodes, patient and economic factors. Comprehensive data has shown a substantial mortality benefit supporting the use of ICDs in-patients with post infarction VT. At present ablation should ideally be considered to have a role as an adjunct to an ICD in-patients with highly symptomatic VT.

As mapping and ablation techniques develop and the accuracy of predicting the long-term outcome improves, the role of RFA may expand, making it the therapy of first choice in a growing proportion of patients. Perhaps with time ICDs may be reserved as the treatment for failed ablation procedures or for prophylactic indications, particularly in parts of the world where widespread implantation of ICDs is financially impractical. Even if ICDs remain justified on the basis of residual uncertainty, the quality of life is likely to be improved by the reduction in discharges resulting from catheter ablation.

## Conclusions

Radiofrequency catheter ablation of patients with highly symptomatic, sustained, monomorphic post infarction VT can be performed with high success rate and acceptable procedural complication rate. The procedure can be successfully applied to a wide spectrum of patients including those with multiple morphologies of VT and hemodynamically unstable VT. The procedure at present requires extensive understanding of anatomical and electrophysiological principles and is prolonged and technically demanding. Its application beyond a few expert centers is dependent upon electrophysiological and technological advances.
